# Laparoscopic rectal surgery in the elderly: clinical outcomes compared to open surgery

**DOI:** 10.1186/1471-2482-13-S1-A1

**Published:** 2013-09-16

**Authors:** Bruno Amato, Rita Compagna, Guido Coretti, Gabriele Vigliotti, Aldo Rocca, Francesca Fappiano, Roberto Rossi, Maurizio Amato, Raffaele Serra, Giovanni Aprea

**Affiliations:** 1 Department of Clinical Medecine and Surgery, University of Naples Federico II, Via S. Pansini,5 – 801311 Napoli, Italy; 2 Department of Medical and Surgical Science -University Magna Gracia of Catanzaro - Viale Europa, Località Germaneto - 88100 Catanzaro, Italy

## Background

The treatment of extraperitoneal cancer of the rectum(ERC) has been greatly improved by the advent of the technique of total mesorectal excision(TME)[1] and the combination of chemo-and radiotherapy in neoadjuvant treatment.

Since the introduction of laparoscopic surgery in the early nineties, the improvements of both the surgical techniques of laparoscopic instruments have allowed us to perform many operations for ERC through a laparoscopic approach.

The aim of this study was to evaluate the short and long-term clinical outcomes in patients treated with Laparoscopic (L.S.) or Open Surgery (O. S.) for the treatment of ERC.

## Materials and methods

Between October 2007 and December 2010 were prospectively collected data of all patients with ERC occurred for the first time, which underwent elective surgery of rectal resection performed by the same operator. Patients with fixed tumors or metastatic disease were excluded. 4 patients (3 pts T3-T4 and 1 pt T-N1) underwent preoperative chemotherapy (Capecitabine, Oxaliplatin) and radiotherapy. Surgery was performed at 6 weeks after discontinuation of radiotherapy. In 5 patients is practiced a temporary ileostomy.

## Results

30 patients had rectal excision for invasive ERC: 15 were treated by laparoscopy and 15 by open procedure. At 3 years, there was no difference of local recurrence and cancer-free survival between L.S. and O.S. groups. The cases treated with laparoscopic approach are subject to a lower morbidity for what concern parameters divided into short and long term[2]. In particular we had a more rapid gastro intestinal function recovery (3 days with L.A. Vs 5,9 days with O.A.), fewer cases of reduction in lung function, fewer days of hospital stay(9,6 days with L.A. Vs 12,2 days with O.A ), fewer cases of wound infection(0 with L.A. Vs 1 with O.A.) and just one case of anastomotic dehiscence with fistula(3 cases in patients treated with O.S.) for patients treated with L.S. Oncological parameters are respected in both techniques, but slightly better in patients who underwent L.S.

## Conclusions

The L.S. can be safely applied to ERC, as oncological principles are respected and postoperative parameters are improved[3] if compared to those of O. S. The laparoscopic technique should therefore always be preferred whenever it is possible.
